# A gene-based SNP resource and linkage map for the copepod *Tigriopus californicus*

**DOI:** 10.1186/1471-2164-12-568

**Published:** 2011-11-21

**Authors:** Brad R Foley, Colin G Rose, Daniel E Rundle, Wai Leong, Gary W Moy, Ronald S Burton, Suzanne Edmands

**Affiliations:** 1 Department of Biological Sciences, University of Southern California, Los Angeles, CA 90089-0371, USA; 2 Windward School, Los Angeles, CA 90066-2104, USA; 3 Department of Organismic and Evolutionary Biology, Harvard University, Cambridge, MA 01730, USA; 4 Marine Biology Research Division, Scripps Institution of Oceanography, University of California, San Diego, La Jolla, CA 92037, USA

## Abstract

**Background:**

As yet, few genomic resources have been developed in crustaceans. This lack is particularly evident in Copepoda, given the extraordinary numerical abundance, and taxonomic and ecological diversity of this group. *Tigriopus californicus *is ideally suited to serve as a genetic model copepod and has been the subject of extensive work in environmental stress and reproductive isolation. Accordingly, we set out to develop a broadly-useful panel of genetic markers and to construct a linkage map dense enough for quantitative trait locus detection in an interval mapping framework for *T. californicus--*a first for copepods.

**Results:**

One hundred and ninety Single Nucleotide Polymorphisms (SNPs) were used to genotype our mapping population of 250 F_2 _larvae. We were able to construct a linkage map with an average intermarker distance of 1.8 cM, and a maximum intermarker distance of 10.3 cM. All markers were assembled into linkage groups, and the 12 linkage groups corresponded to the 12 known chromosomes of *T. californicus*. We estimate a total genome size of 401.0 cM, and a total coverage of 73.7%. Seventy five percent of the mapped markers were detected in 9 additional populations of *T. californicus*. Of available model arthropod genomes, we were able to show more colocalized pairs of homologues between *T. californicus *and the honeybee *Apis mellifera*, than expected by chance, suggesting preserved macrosynteny between Hymenoptera and Copepoda.

**Conclusions:**

Our study provides an abundance of linked markers spanning all chromosomes. Many of these markers are also found in multiple populations of *T. californicus*, and in two other species in the genus. The genomic resource we have developed will enable mapping throughout the geographical range of this species and in closely related species. This linkage map will facilitate genome sequencing, mapping and assembly in an ecologically and taxonomically interesting group for which genomic resources are currently under development.

## Background

Copepods are among the most abundant metazoans on the planet and include more than 11,500 described species [1]. They are ecologically important, spanning a broad range of marine, freshwater and moist terrestrial habitats and serving as major players in global energy transfer, including the largest portion of carbon turnover in the oceanic food web [2]. They are also of economic interest, serving as both prey and parasites of valuable fishery species. Despite their ecological and economic importance, there are few genetic resources developed for copepods, and existing models are likely to be of limited use in this group.

The overwhelming majority of arthropod model systems are insects. Additionally, *Daphnia pulex *(a branchiopod crustacean) has recently been sequenced [3]. However, neither insects nor *Daphnia *are phylogenetically close to copepods. While the precise topology of the arthropod phylogeny is currently debated, the split between Insecta, Copepoda and Branchiopoda is deep in the Cambrian. Shrimp (decapod crustaceans) are somewhat more closely related to copepods and have been the subject of genetic work [e.g. 4, 5] due to their economic importance in the seafood market. However, like other decapods, shrimp have high rates of genomic duplication, complicating genomic analyses and rendering them less useful as models [6-8].

*Tigriopus californicus *is an increasingly attractive genetic model copepod and model crustacean. It has a history as an experimental system for environmental stress, ecotoxicology and speciation [9-12], is easily cultured in the lab, and has an experimentally tractable life cycle. It also has one of the smaller known crustacean genomes--a full order of magnitude smaller than the tiger shrimp *Penaeus monodon *[5].

*T. californicus *has an extensive range along the west coast of North America, from Alaska to Baja California. It is a supralittoral marine harpacticoid copepod, inhabiting splash pools of the rocky shore. It has extremely limited gene flow, with strong genetic differentiation between even geographically close populations [13,14]. Populations from Santa Cruz and San Diego, California are the focus of concerted efforts for the development of genetic resources by several labs. The transcriptomes of the two populations have recently been published [10]. A linkage map comprising 8 microsatellite markers and 45 nuclear SNPs has also recently been constructed [15] with a corrected map length of 484.8 cM, where the 11 linkage groups and one unlinked marker apparently corresponded to the 12 chromosomes of *T. californicus *[16].

A linkage map is a reference point for understanding the genetic basis of phenotypic variation. It is a valuable tool for genome-wide scans of genetic variation, for dissection of QTL (quantitative trait loci) and for the assembly of a physical map. By focusing on developing gene-based markers, our map will facilitate comparative studies using orthologous genes.

We here develop a linkage map between the Santa Cruz and San Diego populations, comprising 190 SNP markers. We validate these markers in 9 additional, geographically distinct populations of *T. californicus*. We also screen the markers in two commonly studied congeners with similar ecology: *T. japonicus*, which is distributed around the Asian Pacific rim, and *T. brevicornis*, which is distributed from Portugal to Nova Scotia around the Northern Atlantic. Many of the markers we develop are also reliably detectable in these two congeners, and will be broadly useful as a community resource for genetic mapping in these species. The development of this linkage map is an essential prelude to the more detailed genomic characterization of a model copepod.

## Methods

### SNP development

The majority of SNPs were identified from 454 cDNA sequence data for the Santa Cruz (SC, 36°57'N, 122°03'W) and San Diego (SD, 32°45'N, 117°15'W) *T. californicus *populations [10]. Sequences were aligned using the gsAssembler (Roche) and visualized in Consed [17]. Diagnostic SC/SD fixed SNPs were chosen using the following criteria: contig > 200bp, minimum of 2 reads for each population, significant sequence alignment to metazoan sequence (BLAST2GO [18]) with an E-value ≤ 10^-3 ^and sufficient conserved flanking regions for priming sites. Another subset of potential SNPs were identified from known gene sequences obtained from Genbank or from unpublished data (R. Burton). For each of the markers with a positive genetic identification, the name of the marker is consistent with the putative-orthologue name, taken as the most-significant alignment using tBlastx at [19]. The one exception is TFAM, where the most significant alignment is to a predicted gene product in *Tribolium castaneum *of the general class of transcription factors which includes TFAM. Subsequent best alignments are to TFAM orthologues in a number of species.

Primers and extension sequences for iPlex high throughput genotyping were developed from this dataset by Jeffrey Conroy at the Genomic Shared Resources, Roswell Park Cancer Institute, Buffalo, NY, using the iPlex Gold software (Sequenom, San Diego CA). Primer and extension sequences are shown in Additional File 1. 201 SNP assays were designed for multiplexing in a total of 7 wells, with a maximum of 30 assays in each well. Sequences ranged from 15-29bp. Within each well, the minimum molecular weight of a sequence was 4567 daltons, and the maximum weight was 8949.9 daltons (Additional File 1). The minimum difference between any two products was 16 daltons. Sequences were scored for molecular weight in a Sequenom mass spectrometer, and SNP identity determined by molecular weight.

SNPs were validated for 5 adult males and 5 adult females from each of the SC and SD lines, and found to be fixed for alternate alleles in each population, with heterozygotes confirmed in 20 F_1 _hybrids. SNPs were also tested in 9 additional populations of *T. californicus *(Table 1), and a single population each of *T*. *brevicornis *and *T. japonicus *(average n = 5). A panel of 46 previously utilized SNPs [15] was also included, including 2 mitochondrial markers to test for contamination.

**Table 1:** Patterns of marker segregation among populations of *T. californicus *and two sister species.

pop (n=)	SD (20)	SC (19)	PA (5)	LMC (4)	RP (5)	CAT (5)	LC (5)	SCI (5)	CAR (5)	AG (10)	WIZ (10)	T.bv (5)	T.jp (5)
amp	190	190	144	148	188	187	189	187	153	153	153	68	92
SD	**0**	190	88	75	108	108	114	120	129	126	131	25	48
SC	*12*	**0**	33	58	79	67	67	57	3	18	20	23	26
PA	*12*	*12*	**25**	34	37	32	32	30	25	28	28	15	20
MC	*12*	*12*	*12*	**15**	17	17	19	22	46	46	46	22	25
RP	*12*	*12*	*12*	*12*	**2**	13	17	27	52	49	51	23	29
CAT	*12*	*12*	*12*	*12*	*12*	**13**	10	23	45	42	43	21	27
LC	*12*	*12*	*12*	*12*	*10*	*10*	**10**	22	42	40	42	23	27
SCI	*11*	*12*	*12*	*12*	*12*	*12*	*12*	**11**	36	32	32	16	27
CAR	*12*	*12*	*12*	*12*	*12*	*12*	*12*	*12*	**21**	5	5	20	20
AG	*12*	*12*	*12*	*12*	*12*	*12*	*10*	*10*	*12*	**9**	0	20	23
WIZ	*12*	*11*	*11*	*12*	*12*	*11*	*12*	*12*	*12*	*12*	**2**	20	23
T.brev	*12*	*12*	*12*	*12*	*11*	*11*	*12*	*12*	*12*	*12*	*12*	**20**	5
T.jp	*12*	*12*	*12*	*12*	*12*	*12*	*5*	*12*	*12*	*11*	*12*	*11*	**18**

Number of fixed differences in SNPs between populations (above diagonal); number of polymorphic SNPs within population (diagonal); number of chromosomes between populations potentially distinguishable by the full panel of 190 SNP markers, including fixed differences and polymorphic alleles (below diagonal).

SD: San Diego, CA, 32°45'N, 117°15'W; SC: Santa Cruz, CA, 36°57'N, 122°03'W; PA: Playa Altamira, Baja California, 28°33'N, 114°05'W; LMC: Los Morros Colorados, Baja California, 29°43'N, 115°18'W; RP: Royal Palms, Palos Verdes CA, 33°42'N, 118°19'W; Cat: Catalina Island, CA, 33°27'N, 118 29'W; LC: Leo Carillo Beach, CA, 34°03'N, 118°56W; SCI: Santa Cruz Island, CA, 34°03'N, 119°34'W; CAR: Carmel, CA, 36°55'N, 121°93W; AG: Aguilar, BC, 48°51'N, 125°08'W; WIZ: Wizard, BC, 48°85'N, 125°16W; T.bv: *Tigriopus brevicornus*; T.jp: *Tigriopus japonicus*.

### DNA extraction

Nauplii (larvae) for genotyping were hatched from late-stage egg sacs isolated in single drops of seawater in a petri plate. The petri plate was flooded with lysis buffer (10 mM Tris pH 8.3, 50 mM KCl, 0.5% Tween 20). Individual nauplii were pipetted in 2-5 μl of buffer to 200 μl PCR tubes containing 10 μl of buffer with 200 μg/ml Proteinase K (final concentration). Tubes were incubated at 65°C for 1 hr followed by 100°C for 15 min and then frozen at -80°C. DNA extraction of adult individuals followed a similar procedure except that individuals were briefly rinsed in diH_2_O and blotted dry on filter paper (egg sacs were gently removed from females) before being frozen at -80°C. Adult samples were subsequently extracted in 50 μl lysis buffer with 200 μl/ml Proteinase K (final concentration). 20 μl of each sample was dried at 60°C for 4 hours before being sent to the Roswell Park Cancer Institute for genotyping, and the remaining 30 μl was retained at -80°C for future work.

### F_2 _mapping population

Upon maturation, males of *T. californicus *perform mate guarding--holding an immature female until she is mature, then mating with her and releasing her. Observing clasped dyads facilitates sexing of immature individuals, and allows the ready collection of virgins. Females are known to mate only a single time in their life, and use stored sperm to fertilize multiple clutches of eggs [20]. Inbred--isofemale--lines are therefore established by isolating a single fertilized female and allowing offspring, including those from overlapping generations, to mate freely.

Isofemale lines were established from SC and SD populations and were maintained in the laboratory for 2.5 years. With a minimum generation time of 23 days, at 20°C, this corresponds to approximately 39 generations of culture. All laboratory stocks were reared in a standard medium of thrice-filtered (40 μm nitex filters) seawater with added growth medium (0.1 g of spirulina and 0.1 g of ground Tetramin per litre of seawater). Crosses between SD females and SC males were conducted in 250 ml beakers containing 200 ml of growth medium. Six replicate crosses consisted each of 50 males and 50 females. Males were discarded after 1 week. Females were removed after 2 weeks, when copepodids (juveniles) became visible. When clasping pairs of F_1 _offspring were observed, the males and females were separated and then united with a partner descending from a different beaker to avoid potential full sibling mating (see Additional File 2). Single pairs comprising an F_1 _female and an F_1 _male were placed in separate petri plates containing 12 ml of culture medium. Males were removed when an egg sac was observed, and females moved to fresh plates as each successive clutch hatched. 250 first instar nauplii were collected for genotyping. In previous experiments, little segregation distortion has been observed in early stage nauplii [15].

### Additional samples for genotyping

In addition to the F_2 _hybrids we also genotyped 19 non-recombinant backcross hybrid adults [(SD female × SC male) F_1 _female × SD male; see Additional File 2]. Because *T. californicus *females do not undergo recombination [16,21] this non-recombinant cross allows confirmation that linkage groups identified from the F_2 _mapping population are on different chromosomes. Lastly, between 5 and 10 adult individuals from 9 other populations of *T. californicus*, as well as *T. brevicornis *and *T. japonicus *were collected for genotyping.

### Mapping

The use of previously mapped SNPs [15] and cytochrome-c [22] enabled us to maintain chromosomal identity from previous work. Chromosomes 1, 2, 5, 7, 8, 9, 10, 11 and 12 were anchored to the linkage groups of Harrison and Edmands [23] and Pritchard et al. [15] (where chromosome 12 corresponds with linkage group D). Our current chromosomes 3, 4 and 6 correspond to the linkage groups of A, B, and C respectively of Pritchard et al. [15] and we were not able to anchor them to Harrison and Edmands [23].

The linkage map was constructed using the JoinMap 3.0 software package [24]. The Kosambi mapping function was used, with an LOD threshold of 1.0, a recombination threshold of 0.4, and default values for all other settings. Correct assignment of markers to chromosomes was confirmed using the non-recombinant backcross genotypes. Several markers exhibited substantial apparent segregation distortion, departing significantly from Hardy Weinberg expectation (χ^2 ^>14, p < 0.001, df = 1). We did not exclude these *a priori *from the analysis. Following construction of the map, we examined the proposed haplotypes and found that, for the 5 most distorted markers we would have to posit recombination in females at levels above 25% between the distorted markers and their nearest neighbors to explain the marker states. As recombination in females is known to not occur at levels >1% [16,21], we excluded these 5 markers from the analysis. We were unable to determine the source of this apparent genotyping error, but one possibility is cryptic, previously unidentified polymorphism.

### Predicted effect of SNPS on protein sequence

Nucleotides that are under lower selective constraint might be expected to have higher substitution rates, and thus may be expected to have more alleles across populations. For all SNPs, we used the probable coding sequences of Barreto et al. [10] to determine whether the SNP was in a translated region of the transcript, and if so, whether or not the SNPs were synonymous. SNP positions and translations were calculated by matching the Sequenom extension sequences against the transcripts of Baretto et al [10] using the Bioperl (v1.6.1) module of Perl (v 5.12.2).

### Synteny with model species

We attempted to find signs of macrosynteny with several assembled arthropod genomes--the insects *Apis mellifera *[25], assembly 4.5, with a 1n = 16; *D. melanogaster *[26] assembly 4.3, with 1n = 5 (commonly the major chromosomal arms are treated as independent linkage groups); and *Tribolium castaneum *[27] assembly 3.0 with a 1n = 12; and the recently sequenced Branchiopod crustacean, *Daphnia pulex *[3] beta 3 release with a 1n = 12. Of these, the insect genomes were the most fully assembled, and we were able to disregard any unanchored scaffolds. In *D. pulex*, there were far more unanchored scaffolds--3804--and we did not exclude any (although the majority of the genome is assembled in the first 50 scaffolds and the majority of our Blast alignments were expected to localize on these).

To identify homologs, we performed sequence alignment searches using tBlastx of the flanking sequence for each of our mapped markers (cDNA contigs for new markers and ESTs for markers from Pritchard et al. [15]) against each reference draft genome assembly, with a threshold of E-value ≤ 10^-3 ^and the default settings. Several sequences had multiple alignments exceeding this threshold, and in these cases we considered only the most significant sequence alignment. The *A. mellifera *amino acid sequences were obtained from[28]. The *T. castaneum *amino acid sequences were obtained from [29], and the chromosomal location of the most significant alignments for both the *A. mellifera *and *T. castaneum *genomes were obtained by querying the Batch Entrez database at [19] with the gi numbers. The *D. melanogaster *v. 4.3 sequences were obtained from Flybase [30]. The *D. pulex *amino acid sequences were obtained from [31] and the scaffold identity of these sequences subsequently obtained by tBlastn to the genomic scaffold obtained at the same site. Where a pair of putative homologs colocalized to a single linkage group in both species, we scored it as a single potential syntenic unit. Where three markers colocalized in this way, we scored 2 alignments (in no case did more than 3 homologs colocalize in both species). We summed all potential syntenic units for each species (Table 2). Significance was determined by randomly permuting the homolog locations relative to the *T. californicus *chromosomal map positions 1000 times in R [32], and counting the number of estimated syntenic units each time.

**Table 2:** Number of pairs of colocalizing loci in both *Tigriopus californicus *and assembled reference genomes.

	linkage groups	n align	n pairs	threshold	p-value range
*Apis mellifera*	16	124	46	43	**0.007-0.002**
*Tribolium castaneum*	10	134	55	61	0.649-0.535
*Drosophila melanogaster*	5	144	91	94	0.563-0.369
*Daphnia pulex*	>3000	72	2	4	0.607-0.321

The number of linkage groups of the draft reference genomes of each species are shown, with the number of identified homologues between *T. californicus *and each of the reference genomes, and the number of homologue pairs which colocalize to a linkage group in both *T. californicus *and the reference. The maximum (threshold) number of colocalized pairs we would expect to see by chance, and the significance of the observed number of colocalized pairs were determined by permutation.

## Results

### SNP development

Of the total of 245 nuclear SNPs we assayed, 190 SNPs were found to be reliable in our genotyping pools, including 38 of the previously developed markers [15]. Only these 190 SNP markers are reported here (Additional File 1). In nauplii, none of these markers were found to be significantly distorted after Bonferroni correction. Primers and extension sequences were found to be widely conserved within populations of *T. californicus*, as well as sister species *T. brevicornis *and *T. japonicus*. Many of the SNPs identified in the SC-SD comparison were polymorphic within other populations (Table 1, diagonal, bold; Additional File 1). The number of fixed differences between populations, as well as segregating polymorphisms within populations, together represent the potential pool of markers for mapping in *T. californicus*. The number of potential markers for use in mapping differences between population pairs appears in the above-diagonal of Table 1.

### Mapping

**Figure 1:**
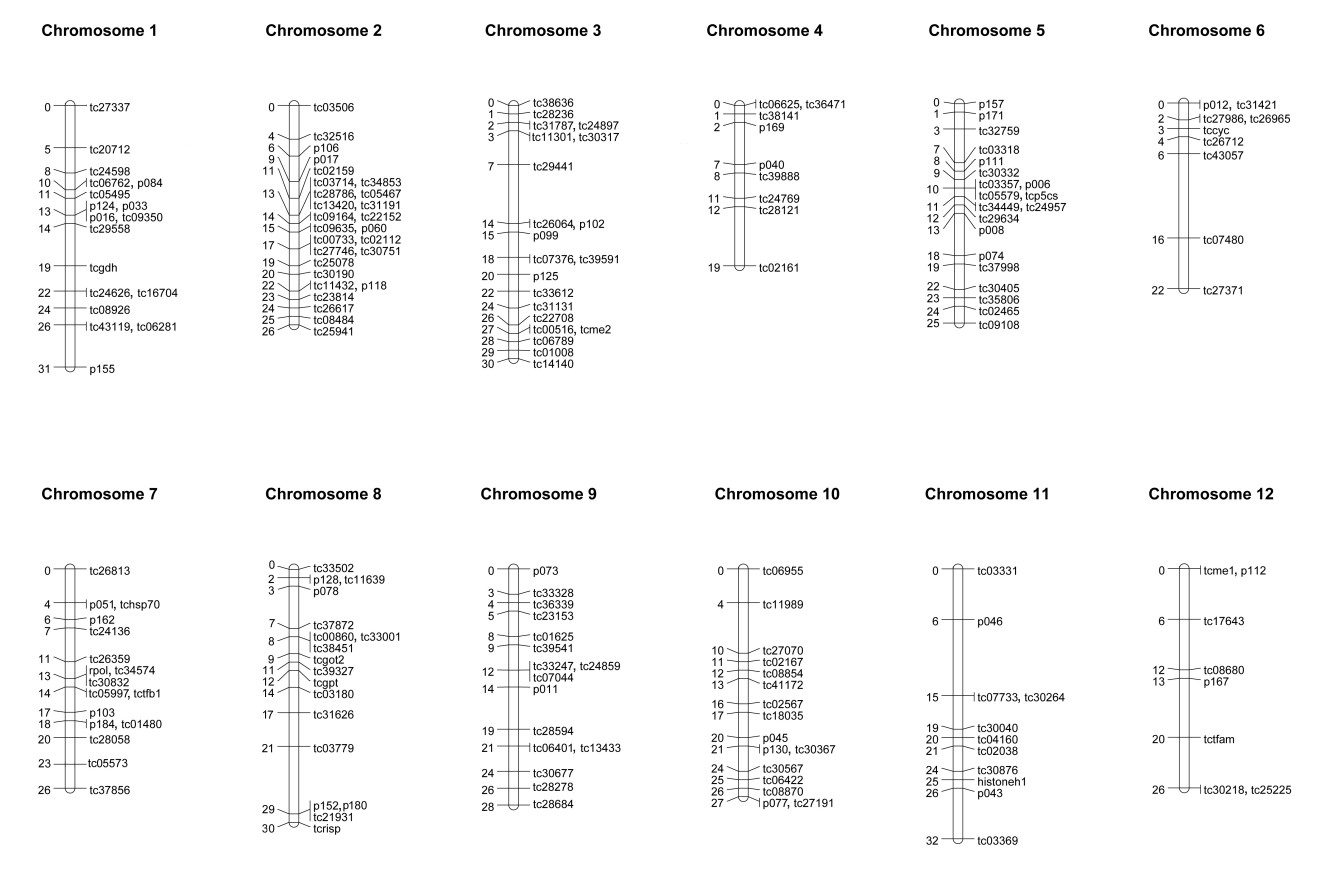
**linkage map of *Tigriopus californicus*, in centimorgans**.

The final assembly consisted of 12 linkage groups, corresponding to the known 12 chromosomes of *T. californicus *(Figure 1). These 12 linkage groups were confirmed by results from the non-recombinant backcross. The average intermarker distance was 1.81 cM, and the maximum distance between adjacent markers was 10.3 cM. The sum of the distances between the markers gives an estimated linkage map length of 323.5 cM long. Applying the suggested correction method [number 4 in 33] to estimate total map length, where L_i _is map length of a given chromosome i, and m_i _is the number of markers for that linkage group, $L_{tot}=\sum_{i}\left(\;L_{i+2}\right)\times \left(m+1\right)∕\left(m-1\right)=401.0\;\text{cM}$. We estimated total coverage following [34]. Given total marker number n = 190, and average intermarker distance d = 1.81 cM, the proportion coverage $c=1-e^{-2dn∕L_{tot}}=0.737$. The recombination rate, given the estimated haploid genome size of 244.5Mb [35,36], corresponds approximately 1.6 cM/Mbp. The sizes of the linkage groups ranged from 19 to 32 cM.

### Predicted effect of SNPs on protein sequence

Of the 190 SNPs used to construct the linkage map, 125 (66%) are predicted to be translated, and of these 125, 101 (79.5%) are synonymous substitutions. Of these 101 loci, there are other synonymous substitutions possible at 80 loci (Additional File 1).

### Synteny with model species

Orthologues to 153 of the 190 *T. californicus *sequences were detected in the reference genomes (Additional File 3). The greatest number of detected orthologues (144) was with reference to the *D. melanogaster *genome, and the lowest (72) was with *D. pulex*. There was evidence of macrosynteny between *T. californicus *and the *A. mellifera *genome. Nine pairs of orthologous genes were found to colocalize on chromosomes in both species, above the maximum of 7 pairs we might expect to find colocalizing by chance. After applying a Bonferroni correction for 4 tests, for each of the reference genomes, the confidence interval for the number of syntenic units shared between the two species is still below the p = 0.0125 threshold for significance as determined by permutation (Table 2, Table 3).

**Table 3:** Chromosomes with colocalizing homologous sequences in *T. californicus *and *A. mellifera*.

*T. californicus *chromosome	*A. mellifera *chromosomes			
1	1(3)	14(2)	-	-
2	4(2)	11(2)	12(2)	15(3)
3	1(2)	6(3)	11(2)	-
4	15(2)	-	-	-
5	1(4)	2(3)	15(2)	-
6	8(3)	-	-	-
7	1(2)	10(3)	11(3)	-
8	1(4)	6(2)	8(3)	10(2)
9	7(2)	11(3)	16(2)	-
10	1(3)	3(2)	4(2)	5(3)

## Discussion

We successfully mapped the *T. californicus *nuclear genome, using 38 previously described SNPs, and 152 novel SNPs, to a total of 190 markers. This corresponds to an average of 1 marker every 1.29 Mbp, and an average intermarker distance of 1.8 cM. All markers were assigned to linkage groups, and the maximum intermarker distance was 10.3 cM. We will thus be able to use these markers to impute genotypes in interval mapping with 95% accuracy or better for all positions in the genome. The 12 linkage groups obtained corresponded to the 12 known chromosomes of *T. californicus*, and most were anchored to linkage groups previously identified with microsatellite and Amplified Fragment Length Polymorphism (AFLP) markers [23]. The total corrected map length of 401.0 cM was somewhat lower than the estimated map length of Pritchard et al. [15], and the estimated total coverage of 73.7% was also somewhat lower than their 75.2% coverage, but this difference in estimates is unsurprising given the much deeper coverage we have achieved in the current study.

All crosses between populations of *T. californicus *from Southern California to British Columbia produce fertile F_1 _progeny [37]. Pairs of populations, however, exhibit increasing F_2 _hybrid breakdown by distance--populations as close as 100 km can have a reduction in fitness of 50% in the F_2 _relative to the parental populations [13,37]. We found no evidence of segregation distortion in the nauplii of our mapping population, however. This is consistent with previous studies which found generally undistorted allele frequencies in the early larval stage, while adults had allele frequencies which strongly departed from expected frequencies [15,38].

Primer sequences used for polymerase chain reaction DNA amplifications were largely conserved between populations. In only one population (PA) did more than 5 primer sets fail to amplify a product, and we found many SNPs segregating within and between populations. The elevated differentiation between PA and the Southern Californian populations was not unexpected, as Baja populations of *T. californicus *have been found to be very divergent in mitochondrial haplotype [13], and to exhibit high rates of hybrid breakdown when crossed with northern populations [39]. Many of our primers also amplified products in the sister species *T. japonicus *and *T. brevicornis*. The SNP markers we have characterized here, therefore will be generally useful for QTL mapping across the entire geographic range of *T. californicus*. For all but a few population pairs, it will be possible to construct mapping lines and distinguish every chromosome in a non-recombinant backcross design.

Additional SNPs may be found to segregate in these populations, since they were only genotyped for the known SD and SC SNPs (or SC, SD and a third population in the case of the SNPs from Pritchard et al. [15]). Only 20 of the SNP markers were in coding regions at twofold degenerate sites, suggesting as many as 89.5% of the SNPs may be under relaxed selective constraint. Populations will thus need to be characterized for alternate alleles for mapping purposes. The known level of triallely of SNPs is very low in *T. californicus*, however. V. Pritchard (pers. comm) found that between San Diego, Santa Cruz and Punta Baja (Baja California), levels of triallely are approximately 2% at SNP sites across the genome. These 3 populations are deeply divergent within *T. californicus*, suggesting that within-species at least, there will be few uncharacterized alleles.

Compared with the other arthropods, the recombination rate of *T. californicus *(1.6 cM/Mbp) is low [reviewed in 39], but is more than sufficient for mapping in F_2_, backcross and other linkage mapping approaches. It is similar to that of Diptera(mean = 1.03 cM/Mbp) and shrimp *Penaeus monodon *(mean = 1.01 cM/Mbp)[5], but it is approximately 21% the estimated recombination rate of *Daphnia pulex *at 7.52 cM/Mbp [40]. It is much lower than Hymenoptera (mean = 7.12 cM/Mbp), and somewhat lower than the more typical arthropod recombination rate of Coleoptera (mean = 2.48 cM/Mbp) [41]. It is known that recombination rates in groups with alternate life histories are under selection--for instance, social vs. solitary Hymenoptera have greatly elevated recombination rates. The relatively high recombination rate of *D. pulex *compared to shrimp and *T. californicus *is possibly due to its reproductive mode--many generations of parthenogenesis, punctuated by episodic sex, may have consequences on the evolution of recombination rate [42]. Another reason for the relatively low recombination rate might be that recombination occurs only in males in *T. californicus *[16,21].

Additionally, perhaps surprisingly, there was some evidence of macrosynteny between *A. mellifera *and *T. californicus*, although there was no one-to-one correspondence between chromosomes. No evidence of synteny was found *T. californicus *and *D. pulex *or *D. melanogaster*; however, this may be because these genomes have undergone higher rates of chromosomal rearrangements. It was reported that no evidence of synteny was found between *D. pulex *and *D. melanogaster *either [40]. In fact, Critescu et al. [40] suggest--based on the rearrangement rate of *Drosophila*--that synteny should only be apparent between crustaceans and insects at scales smaller than 100 kb. It may be that Hymenoptera and Copepoda have lower rates of large-scale chromosomal fission and fusion than *Drosophila *and the other assayed arthropod lineages.

## Conclusion

We have mapped the *T. californicus *genome to a level of coverage that will enable common mapping techniques such as nonrecombinant backcross, and F_2 _or Recombinant Inbred Line mapping--greatly expanding the possible scope of work in this species. The markers and map developed will potentially be useful more broadly in the genus *Tigriopus*. The phylogenetic position of *T. californicus*, its compact genome size, and its tractability in the lab have all positioned it as an excellent model crustacean system. The continued development of genomic tools such as this linkage map will enable future exciting work in this species.

## Abbreviations used

*A. mellifera*: *Apis mellifera; D. pulex*: *Daphnia pulex*; *P. monodon*: *Penaeus monodon*; *T. castaneum*: *Tribolium castaneum*; QTL: Quantitative Trait Locus; SNP: Single Nucleotide Polymorphism; *T. californicus/japonicus/brevicornis*: *Tigriopus spp.; *cDNA: coding DNA. Populations: AG: Aguilar, BC, 48°51'N, 125°08'W; CAR: Carmel, CA, 36°55'N, 121°93W; Cat: Catalina Island, CA, 33°27'N, 118 29'W; LC: Leo Carillo Beach, CA, 34°03'N, 118°56W; LMC: Los Morros Colorados, Baja California, 29°43'N, 115°18'W; PA: Playa Altamira, Baja California, 31°52'N, 116°40'W; RP: Royal Palms, Palos Verdes CA, 33°42'N, 118°19'W; SC: Santa Cruz, CA 36°57'N, 122°03'W; SD: San Diego, CA, 32°45'N, 117°15'W; SCI: Santa Cruz Island, CA, 34°03'N, 119°34'W; WIZ: Wizard, BC, 48°85'N, 125°16W.

## Authors' contributions

SE designed the study, assisted with crosses, oversaw analyses and contributed to the final written manuscript. GWM and RSB generated transcriptome data. CGR developed the SNP assays, constructed the linkage map and assisted with crosses. DER and WL assisted with crosses. BRF performed all post-map analyses and wrote the manuscript. All authors contributed comments and approved the manuscript prior to submission.

## Supplementary Material

Additional File 1 **"Table of markers used to create the linkage map**. Showing calculated map positions, forward and reverse primer sequences, extension sequences with associated SD and SC alleles. Marker genotypes of a wide geographic sampling of populations, as well as sister species, are shown. Also shown is the sequence description from the most significant alignment obtained by Blast search (GenBank). Inferred San Diego and Santa Cruz triplet codons are shown, with the SNP position and amino acid translation. If SNPs are predicted to be synonymous, the number of additional synonymous alleles at the SNP site is indicated." Click here for file

Additional File 2 **"Crossing scheme between San Diego (SD) and Santa Cruz (SC) to generate F2 and non-recombinant backcross (nrBC) mapping lines in *Tigriopus californicus***." Click here for file

Additional File 3 **"Table of most significant tBlastx alignments (E-value ≤ 10^-3^) between *T. californicus *sequences and the reference draft genome assemblies of model species *Apis mellifera*, *Tribolium castaneum*, *Drosophila melanogaster *and *Daphnia pulex***. For each significant alignment are shown the gi number, the E-value of the alignment, and the linkage information of the homologue. Click here for file
