# Supplementary figures and images for: A gene-based SNP resource and linkage map for the copepod Tigriopus californicus
Source: BMC Genomics. 2011 Nov 21;12:568. doi: 10.1186/1471-2164-12-568 (PMC3298550; doi:10.1186/1471-2164-12-568)

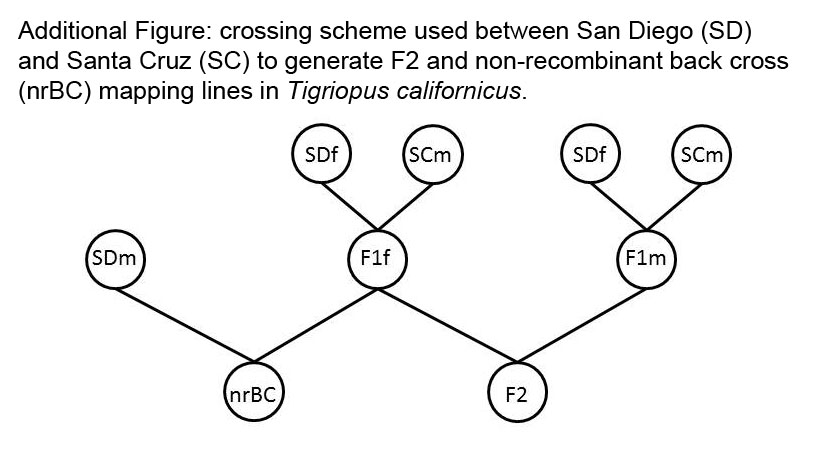

Supplement: Additional File 2 — "Crossing scheme between San Diego (SD) and Santa Cruz (SC) to generate F2 and non-recombinant backcross (nrBC) mapping lines in Tigriopus californicus." [file 1471-2164-12-568-S2.JPEG]
